# Diverse Frontoparietal Connectivity Supports Semantic Prediction and Integration in Sentence Comprehension

**DOI:** 10.1523/JNEUROSCI.1404-24.2024

**Published:** 2024-11-12

**Authors:** Yaji He, Ximing Shao, Chang Liu, Chen Fan, Elizabeth Jefferies, Meichao Zhang, Xiaoqing Li

**Affiliations:** ^1^CAS Key Laboratory of Behavioral Science, Institute of Psychology, Chinese Academy of Sciences, Beijing 100101, China; ^2^Department of Psychology, University of Chinese Academy of Sciences, Beijing 101408, China; ^3^Department of Psychology, York Neuroimaging Centre, University of York, York YO10 5DD, United Kingdom

**Keywords:** language comprehension, prediction, integration, frontoparietal cortices, functional connectivity

## Abstract

Predictive processing in the parietal, temporal, frontal, and sensory cortex allows us to anticipate future meanings to maximize the efficiency of language comprehension, with the temporoparietal junction (TPJ) and inferior frontal gyrus (IFG) thought to be situated toward the top of a predictive hierarchy. Although the regions underpinning this fundamental brain function are well-documented, it remains unclear how they interact to achieve efficient comprehension. To this end, we recorded functional magnetic resonance imaging (fMRI) in 22 participants (11 males) while they comprehended sentences presented part by part, in which we manipulated the constraint provided by sentential contexts on upcoming semantic information. Using this paradigm, we examined the connectivity patterns of bilateral TPJ and IFG during anticipatory phases (i.e., before the onset of targets) and integration phases (i.e., after the onset of targets). When upcoming semantic content was highly predictable in strong constraint contexts, both the left TPJ and bilateral IFG showed stronger visual coupling, while the right TPJ showed stronger connectivity with regions within control, default mode, and visual networks, including the IFG, parahippocampal gyrus, posterior cingulate, and fusiform gyrus. These connectivity patterns were weaker when predicted semantic content appeared, in line with predictive coding theory. Conversely, for less-predictable content, these connectivity patterns were stronger during the integration phase. Overall, these results suggest that both top-down semantic prediction and bottom-up integration during predictive processing are supported by flexible coupling of frontoparietal regions with control, memory, and sensory systems.

## Significance Statement

Recent work has revealed the neural basis of predictive language comprehension. However, it remains unclear how brain regions change their connectivity dynamically to support comprehension in highly predictive and less predictive contexts. Here, we show that stronger frontoparietal connectivity with cognitive control, memory, and sensory areas supports top-down prediction generation in strong constraint contexts; these connectivity patterns are reduced when the anticipated information appears. This pattern is reversed when upcoming sensory input is unpredictable; connectivity is stronger after word inputs have been presented, allowing semantic integration with preceding low-constraint context. Our findings suggest that both top-down semantic prediction and bottom-up semantic integration in language comprehension rely upon diverse functional coupling of higher-order frontoparietal regions with other brain systems.

## Introduction

The human brain is remarkably efficient in language comprehension, capable of actively predicting upcoming semantic content (Kuperberg and Jaeger, 2016; Heilbron et al., 2022). This predictive processing relies upon two inextricably related processes: anticipatory processing of future semantic information based on available context and retrieved knowledge and integration of new inputs with a representation of preceding context, which includes top-down predictions generated from this context (Bonhage et al., 2015; Weber et al., 2016; Shao et al., 2022). According to the predictive coding theory (K. Friston, 2010), when new sensory inputs are consistent with predictions, the bottom-up processing demands of this input and the communication of sensory systems with regions higher in the processing hierarchy are reduced due to suppressed feed-forward propagation of predictable information. This is reflected in reduced cortical activity in high versus low predictable contexts (Baumgaertner et al., 2002; Shao et al., 2022), suggesting a facilitation effect of prediction on the efficiency of language comprehension.

**Figure 1.**
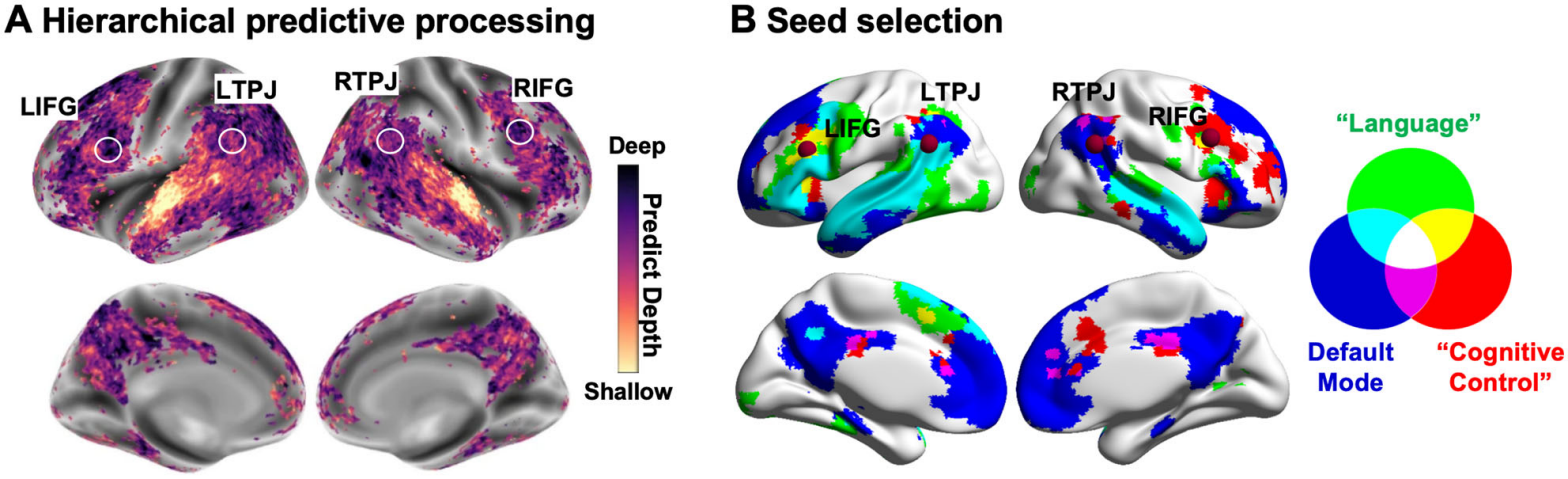
***A***, Organization of hierarchical predictive processing revealed by Caucheteux et al. (2023), utilizing a publicly available fMRI dataset of 345 individuals listening to 27 spoken stories in English. Only significant voxels are color-coded. The dark regions represent deep forecast representations, while the light regions indicate shallow forecast representations. The white circles correspond to the seed regions selected in our analysis, located toward the top of this predictive hierarchy. ***B***, The TPJ and IFG, situated at the top of this predictive processing hierarchy, overlap with the DMN (in blue) and “cognitive control network” (in red) from a term-based meta-analysis using Neurosynth, respectively. Both networks highly overlap with the “language network from a term-based meta-analysis using Neurosynth, highlighting the importance of the regions within these networks in supporting language processing. Our ROIs in bilateral IFG (MNI coordinates: left IFG at −44 22 24 and right IFG at 46 18 32) and TPJ (MNI coordinates: ± 54 −54 28) therefore were selected from the meta-analytic cognitive control network and DMN as seed regions to drive the PPI analyses (radius = 6 mm). L, left hemisphere; R, right hemisphere. Note: Panel ***A*** is adapted under a Creative Commons Attribution 4.0 International License (CC BY 4.0).

Recent work already revealed widely distributed yet hierarchical brain regions subserving predictive language comprehension (Schmitt et al., 2019; Caucheteux et al., 2023), including cognitive control areas of the inferior frontal and supramarginal gyrus (SMG), memory stores of the temporal gyri, integration hubs of parietal sites, and sensory cortices (Fig. 1*A*). The frontoparietal cortices, specifically inferior frontal gyrus (IFG) and temporoparietal junction (TPJ), occupy a prominent position at the top of the hierarchical neural networks, supporting the prediction of contextual-level semantic contents (Schmitt et al., 2019; Caucheteux et al., 2023). Semantic prediction is considered to be generated in a top-down manner (Lewis and Bastiaansen, 2015; Kuperberg and Jaeger, 2016). In this process, IFG plays a crucial role, not only receiving bottom-up input for higher-order computation/unification (Hagoort, 2013) but also mediating top-down controlled semantic processing (Jackson, 2021; Zhang et al., 2021). TPJ is another crucial hub in predictive processing, which supports information integration as sensory information unfolds over time (Lin et al., 2018; Matchin et al., 2019; Lanzoni et al., 2020). Its critical role in integration fits well with the observation that default mode network (DMN), topologically located at the top of cortical hierarchy, underlies the integration of diverse streams of information (Margulies et al., 2016; Smallwood et al., 2021). Although previous work has described this predictive processing hierarchy, we still lack a detailed mechanistic account of how these brain areas interact to subserve the anticipatory and integration processes that arise during predictive language comprehension.

Communication between higher-order and lower-level brain areas is necessary for prediction. However, connectivity patterns (routes or strength) between these regions might vary across anticipatory and integration phases as distinct cognitive processes might be involved in these phases (Bonhage et al., 2015; Weber et al., 2016; Shao et al., 2022). When upcoming semantic content is predictable from its previous context, stronger connectivity between frontoparietal cortices and control, memory, and sensory systems might be important to support the generation and implementation of top-down predictions. This connectivity pattern might decrease when predictions are confirmed by the subsequent sensory inputs, due to reduced demands on sensory processing and the suppression of feed-forward propagation (K. Friston, 2010). In unpredictable contexts, however, the connectivity of higher- with lower-hierarchical predictive brain areas might be weaker before the appearance of target information, since it is hard to generate a specific context-relevant semantic prediction; however, when unpredictable target information actually appears, stronger connectivity of higher-order frontoparietal cortices with other systems, especially sensory regions, might support coherent comprehension during bottom-up integrative processing.

To test these hypotheses, we manipulated the semantic constraints of sentential contexts on upcoming semantic content (Strong vs Weak) and examined both anticipatory and integration phases. Given the importance of IFG and TPJ within frontoparietal cortices in semantic prediction (Siman-Tov et al., 2019; Caucheteux et al., 2023), we aimed to explore (1) the changes in the connectivity patterns of these core regions depending on the strength of prediction that is possible for a sentence and (2) how these patterns of connectivity vary across semantic prediction and integration phases.

## Materials and Methods

### Participants

A published dataset of 22 participants (age range, 19–25; 11 males) was used in this study (Shao et al., 2022). All were right-handed native Mandarin speakers, with normal or corrected-to-normal vision. None had any history of neurological impairment, diagnosis of learning difficulty, or psychiatric illness. All provided written informed consent prior to taking part and received monetary compensation for their time. Ethical approval was obtained from the ethics committee of the Institute of Psychology, Chinese Academy of Sciences.

### Materials

**Figure 2.**
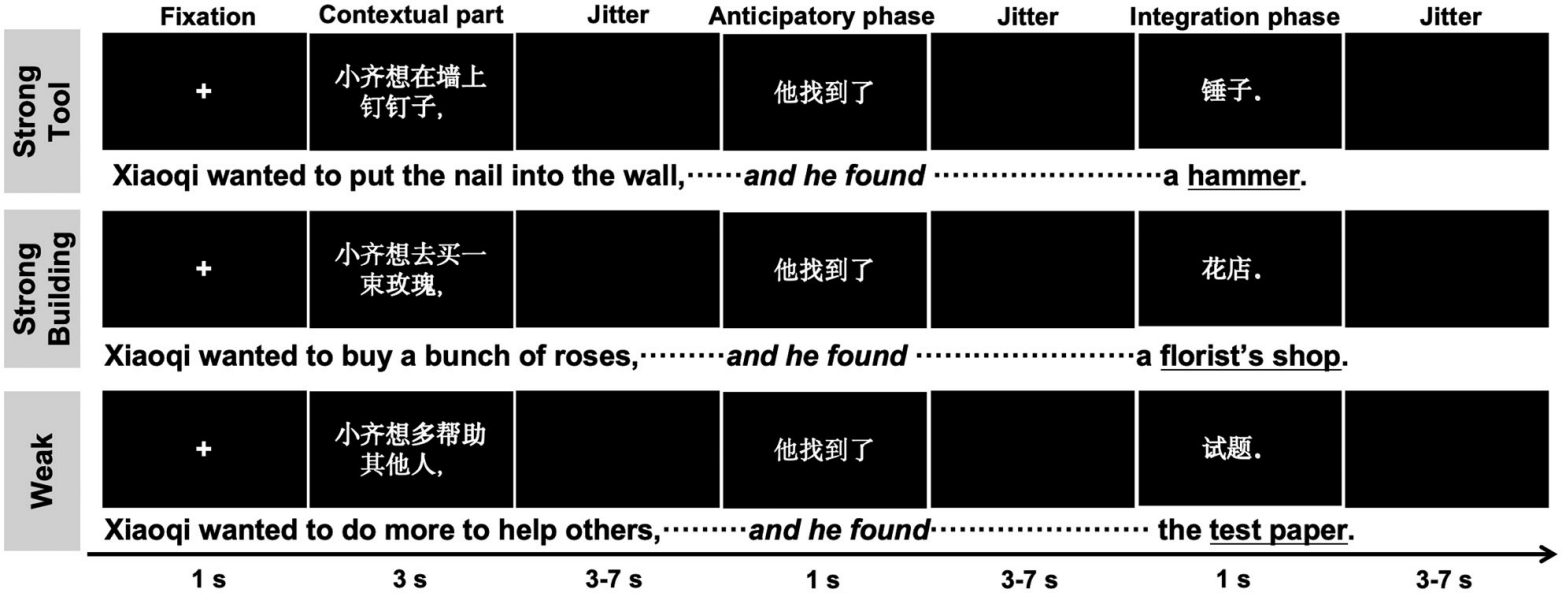
Task illustration. Participants were asked to read and comprehend the Strong Tool, Strong Building, and Weak semantic constraint sentences. The italicized words indicate the pronoun + critical verbs (i.e., anticipatory phase), and the underlined words indicate the critical nouns (i.e., integration phase). Results of two cloze probability tasks for the predictability of critical nouns and the importance of critical verbs are presented in Extended Data Figure 2-1. Psycholinguistic properties of critical nouns are detailed in Extended Data Figure 2-2.

Twenty-nine sets of sentences in Mandarin Chinese were designed to manipulate the semantic constraint that affects predictive processing during comprehension, with a strong contextual constraint leading to a strong prediction of either tool- [strong constraint tool (Strong Tool)] or building-related semantic information [strong constraint building (Strong Building)] and a weak contextual constraint with a weak prediction of upcoming semantic information [weak constraint (Weak)]. The syntactic structure of these sentences was kept consistent across conditions, with each sentence consisting of two subclauses: the first clause set a communication background, while the second clause followed the structure of “pronoun + transitive verb + critical noun,” in which the verb plays an essential role in the formation of strong prediction of the upcoming critical nouns based on the preceding context regard to the completion of predicate–argument structure (Li et al., 2017, 2020). For example, “Xiaoqi wanted to put the nail into the wall, he found a hammer.” The “critical nouns” were presented at the end of each sentence, with this noun always being the best completion of the preceding strong contextual constrain (i.e., a noun of tool or building), and an inanimate, yet neither a tool nor building, noun (e.g., skateboard and sofa) in the Weak condition, yet still being the best completion of the Weak constraint condition (see Fig. 2). In this way, the noun targets in the Weak condition would not induce semantic violations with its preceding contexts during comprehension.

10.1523/JNEUROSCI.1404-24.2024.f2-1 Figure 2-1 Cloze probability (CP) of the Critical Nouns (and Other Completed Words) in the three Experimental Conditions. “*Critical nouns*” indicate the critical nouns used in the experimental sentences of corresponding experimental condition, “*tool-nouns*” indicate all of the completed nouns that belong to the tool category, and “*building-nouns*” indicate all of the completed nouns that belong to the building category. “*CP of pronoun* *+* *verb*” indicates the averaged CP of the critical verbs and the pronouns immediately preceding these verbs. “*N.A.*” indicates that the corresponding CP value is not applicable, as the pronoun and verb have already been presented at the “Preceding Critical Noun” position. For the Weak-constraint condition, the critical nouns were the best completion in Test 2. Download Figure 2-1, DOC file.

10.1523/JNEUROSCI.1404-24.2024.f2-2 Figure 2-2 The linguistic properties of the critical nouns in the end of sentences. Download Figure 2-2, DOC file.

To validate the importance of critical verbs and the degree of predictability of the critical nouns, an independent dataset of 32 participants was recruited to perform two cloze probability tasks by presenting the sentence until the critical verbs (Test 1; e.g., Xiaoqi wanted to put the nail into the wall, he _____) or critical nouns (Test 2; e.g., Xiaoqi wanted to put the nail into the wall, he found ____). In both of these tests, the predictability of critical nouns in both the Strong Tool and Strong Building conditions was significantly higher than that of the Weak condition (*p* < 0.001), and there was no significant difference between the two strong constraint conditions (*p* > 0.13; Extended Data Fig. 2-1). Moreover, the difference score of critical-noun predictability (i.e., Strong Tool minus WEAK_best completion_ or Strong Building minus WEAK_best completion_) was significantly larger in Test 2 than that in Test 1 (all *p* < 0.001). These results suggest (1) the validity of our manipulation of semantic constraint and (2) the importance of the critical verb in forming a strong semantic prediction of upcoming critical nouns in strong semantic constraint conditions. In addition, for each set of sentences, the pronoun and transitive verb preceding the critical word were exactly the same across the Strong Building, Strong Tool, and Weak constraint conditions (see Fig. 2). Furthermore, the psycholinguistic properties of the critical nouns (Extended Data Fig. 2-2), including word frequency, word length, and stroke number, were well-matched across the three conditions; the tool-nouns were also rated highest in operability and imageability than that of building-nouns and less-predictable nouns (i.e., in the Weak condition), in line with the semantic features of tool-nouns and indicating a successful manipulation of tool-nouns. These materials were the same as those used in our previous study [for details, see Shao et al. (2022)].

### Procedure

Participants were asked to read the sentences for comprehension, which were projected in white font with a size of 20 points onto a black screen. As shown in Figure 2, each trial started with a fixation cross (1 s) in the center of the screen, followed by the sentences presented in a part-by-part manner. The contextual part was presented for 3 s, followed by two subsequent segments, the “pronoun + critical verbs” and “critical nouns” with each part lasting 1 s. These three parts were sequentially presented after a jittered fixation interval lasting 3–7 s. An intertrial interval of 3–7 s was used to eliminate the possibility of BOLD responses from one event being impacted by any residual BOLD response from the previous stimulus. Twenty-nine sets of experimental sentences (87 experimental sentences in total) and 21 filler sentences were allocated to three runs, with each run lasting 16 min. To ensure that participants maintained attention to the presented stimuli, they were asked to press a button if they noticed that the noun at the end of the sentence was semantically violated with its preceding context. Out of the 21 filler sentences, 18 contain semantic violations, with each run including 6 of these sentences. In addition, trials were presented in a pseudorandom order to ensure trials from the same experimental condition were not consecutively presented more than three times. Before entering the scanner, participants completed a brief practice of reading sentences silently and responding to catch trials to ensure a full understanding of the task requirements.

### Neuroimaging data acquisition and preprocessing

Structural and functional data were acquired using a 3T GE Discovery MR750 scanner. Structural images were obtained using a 3D spoiled gradient recall pulse sequence with the following parameters: echo time (TE), minimum full; inversion time, 450 ms; field of view, 256 × 256 mm²; flip angle, 12°; matrix size, 256 × 256; voxel size, 1 × 1 × 1 mm³; 192 slices; and slice thickness, 1 mm. The task-based activity was recorded using a gradient-echo EPI sequence: repetition time (TR), 2000 ms; TE, 30 ms; field of view, 224 × 224 mm²; flip angle, 90°; matrix size 64 × 64; voxel size, 3.5 × 3.5 × 3.5 mm³; 33 slices; and slice thickness, 3.5 mm.

Preprocessing was performed using the Data Processing Assistant for Resting-State fMRI (DPARSF, http://rfmri.org/DPARSF) toolbox (Yan and Zang, 2010) based on SPM12 (https://www.fil.ion.ucl.ac.uk/spm/). We removed the first five time points of each run for steady-state magnetization. Then, functional images were slice-time corrected. After realigning to the midvolume in the time series to correct for head motion, the images were coregistered with anatomical images. The anatomical images were segmented into gray and white matter, and the spatial normalization parameters acquired during this step were used to normalize the functional images. Finally, the images were smoothed with a 6 mm FWHM Gaussian kernel.

### Seed selection

Our analysis focused on understanding how higher-order frontoparietal cortices interact with other brain areas to support semantic prediction and integration during language comprehension. Frontoparietal cortices, particularly IFG and TPJ, have been shown to play an important role in predicting contextual semantic representations (Caucheteux et al., 2023). IFG is considered a key site for semantic prediction (Fuster and Bressler, 2015; Willems et al., 2016; Grisoni, 2022), which exhibits consistent activation across various contrasts designed to evaluate semantic prediction (Rothermich and Kotz, 2013; Weber et al., 2016; Shain et al., 2020; Shao et al., 2022), and is responsible for top-down control of semantic retrieval/selection and unification (Hagoort, 2013; Jackson, 2021; Zhang et al., 2021, 2022). TPJ is another critical region for semantic predictive processing (Schmitt et al., 2019; Masina et al., 2022; Caucheteux et al., 2023), which underlies the integration of information into more meaningful and complex representations (Feng et al., 2015; Lin et al., 2018; Lanzoni et al., 2020), and consequently it is important for updating internal contextual representation as sensory inputs unfolding (Geng and Vossel, 2013). Its critical role in integration fits well with an emerging topographical view of DMN, highlighting its role in the integration of diverse information (Smallwood et al., 2021).

To avoid the double-dipping problem (i.e., using the same dataset for both hypothesis generation and testing; Kriegeskorte et al., 2009) and enhance the reliability and reproducibility of our findings, we selected our seeds based on prior research rather than our own data. Four seeds were therefore defined based on the peak activation within TPJ and IFG from DMN (Andrews-Hanna et al., 2010) and meta-analytic cognitive control network (using Neurosynth), respectively. Both networks highly overlap with the meta-analytic “language network” (Fig. 1*B*), further highlighting the importance of regions within these networks in supporting language comprehension. (1) The left and right TPJ (MNI coordinates, ±54 −54 28) seeds fell within the DMN as defined by Yeo et al. (2011) and corresponded to the peak coordinates in TPJ identified in intrinsic connectivity analysis of DMN architecture conducted by Andrews-Hanna et al. (2010). The TPJ seeds were close to the peak activation in TPJ showing a stronger response during semantic integration (Feng et al., 2015; Matchin et al., 2019; Lanzoni et al., 2020; Shao et al., 2022) and to the TPJ situated at the top of predictive processing hierarchy that supports semantic prediction (Schmitt et al., 2019; Caucheteux et al., 2023). (2) The left (MNI coordinate, −44 22 24) and right IFG (MNI coordinate, 46 18 32) seeds were defined by using the search term “cognitive control” in Neurosynth (Yarkoni et al., 2011), which completely fell within the frontoparietal control network (FPN) and outside the somatomotor (SM) network as defined by Yeo et al. (2011), and were also located at the top of the hierarchical neural networks associated with a semantic prediction (Caucheteux et al., 2023). The IFG seed region was close to the peak response in IFG for controlled semantic retrieval identified by both task-based and meta-analyses of neuroimaging studies of semantic control (Noonan et al., 2013; Jackson, 2021; Zhang et al., 2021), which, like our seed, was located within FPN. The IFG seeds were also close to the sites in IFG supporting combinatorial semantic processing (Zhu et al., 2012; Schell et al., 2017) and active semantic prediction (Wu et al., 2012; Willems et al., 2016; Siman-Tov et al., 2019; Shao et al., 2022).

### Psychophysiological interaction (PPI) analysis

These seeds were created based on their coordinates using a generalized psychophysiological interaction (gPPI) toolbox by a 6 mm radius, and the time series within these seeds were extracted after the BOLD time series were preprocessed in order to create physiological variables for each participant. We then ran a separate gPPI model for each of the four selected seeds. Compared to the standard PPI analysis implemented in SPM, gPPI provides a superior performance in model fitting (McLaren et al., 2012). Most importantly, this method allowed us to analyze the connectivity across multiple experimental conditions within a single model (McLaren et al., 2012).

At the first level, the preprocessed time-series data were modeled using a generalized linear model for each participant, which included the task regressors for each of the three experimental conditions (i.e., Strong Tool, Strong Building, and Weak semantic constraint) as well as the filler sentences at both the anticipatory (i.e., pronoun + the transitive verb part; duration, 1 s) and integrative processing phases (i.e., the critical noun; duration, 1 s), a PPI term for each of the three main experimental conditions during each phase, the time series of the seed, and the contextual parts for each of the three experimental conditions and filler sentences (i.e., first subclause; duration, 3 s). In addition, six motion parameters were included in the model as regressors of no interest.

At the second level, we conducted a whole-brain group analysis to examine the main effects of the processing phase (anticipation vs integration), semantic constraint (Strong Building vs Strong Tool vs Weak), and all two-way interaction terms. The threshold was set at *p* < 0.005 uncorrected at the voxel-wise level and *p* < 0.05 with FEW correction at the cluster level (Lordier et al., 2019). All *p*-values were Bonferroni corrected for the number of seeds; the *p*-value accepted as significant was therefore *p* < 0.0125.

### Data and code availability statement

Neuroimaging data at the group-level statistical *F* maps are openly available in NeuroVault at https://identifiers.org/neurovault.collection:17733. Script for the task and supporting information are accessible in the Open Science Framework at https://osf.io/3hjwm/. The conditions of our ethical approval do not permit public archiving of the raw data because participants did not provide sufficient consent. Researchers who wish to access the data should contact the corresponding authors, X.L. or M.Z., data will be released to researchers when this is possible under the terms of the General Data Protection Regulation.

## Results

Our behavioral results showed that participants detected 91.6 ± 7.6% (mean ± SD) of semantically violated catch trials, suggesting that they were paying attention to the inputs presented on the screen. Next, our study set out to examine whether and how the connectivity patterns of higher-order frontoparietal cortices support semantic prediction and integration during language comprehension. A separate PPI model was conducted for each of the selected TPJ and IFG seeds within frontoparietal cortices, examining the main effects of the processing phase (anticipation vs integration) and semantic constraint (Strong Building vs Strong Tool vs Weak) and all the interaction terms between these two factors on connectivity. The patterns of TPJ and IFG connectivity associated with these effects are described below and summarized in Table 1. We also present the overlap of each connectivity map with the intrinsic connectivity networks defined by Yeo et al. (2011) in Table 2.

**Table 1.** Peak MNI coordinates resulting from the interaction between the processing phase and semantic constraint in connectivity analyses of TPJ and IFG seeds

Seed	Connectivity	*x*	*y*	*z*	Voxel	*F*	*p*-FWE
Right TPJ	Right PHG/fusiform	18	−24	−15	2,714	35.81	<0.001
	Left SMG	−66	−21	18	255	19.09	0.002
	Right SMG	69	−18	21	190	20.66	0.011
Left TPJ	Left MOG/lingual	−15	−90	−3	596	15.68	<0.001
	Right Hippocampus	18	−24	−12	186	26.85	0.012
Left IFG	Left lingual	−21	−63	−6	551	21.75	<0.001
	Right fusiform	27	−63	−3	282	16.03	0.001
Right IFG	Right lingual	3	−78	−9	1,817	29.27	<0.001

Abbreviations: PHG, parahippocampal gyrus; SMG, supramarginal gyrus; MOG, middle occipital gyrus.

**Table 2.** Percentage overlap of connectivity interaction effects for TPJ and IFG seeds with the seven large-scale intrinsic connectivity networks defined by Yeo et al. (2011)^a^

Seed	VN	SM	DAN	VAN	LB	FPN	DMN
Right TPJ	33.01	19.96	2.59	29.13	0.43	8.41	6.47
Left TPJ	94.92	0	3.39	0	0	0	1.69
Left IFG	97.66	0	0	0	0	0	2.34
Right IFG	68.82	0	4.21	0	0	2.25	24.72

a The percentage of voxels in the identified cluster that fell within the seven large-scale networks defined by Yeo et al. (2011), disregarding voxels that did not fall within any of the Yeo networks. VN, visual network; SM, somatomotor network; DAN, dorsal attention network; VAN, ventral attention network; LB, limbic network; FPN, frontoparietal control network; DMN, default mode network.

Given our aim was to understand how connectivity patterns of TPJ and IFG within higher-order frontoparietal cortices change with the availability of semantic prediction in different phases of a sentence, we therefore focused on the interaction effects between processing phase and semantic constraint. Additionally, we report the main effects of these experimental manipulations on connectivity, as well as the main and interaction effects of these two factors for univariate analyses in the supporting information (https://osf.io/3hjwm/).

### Results for the right TPJ

**Figure 3.**
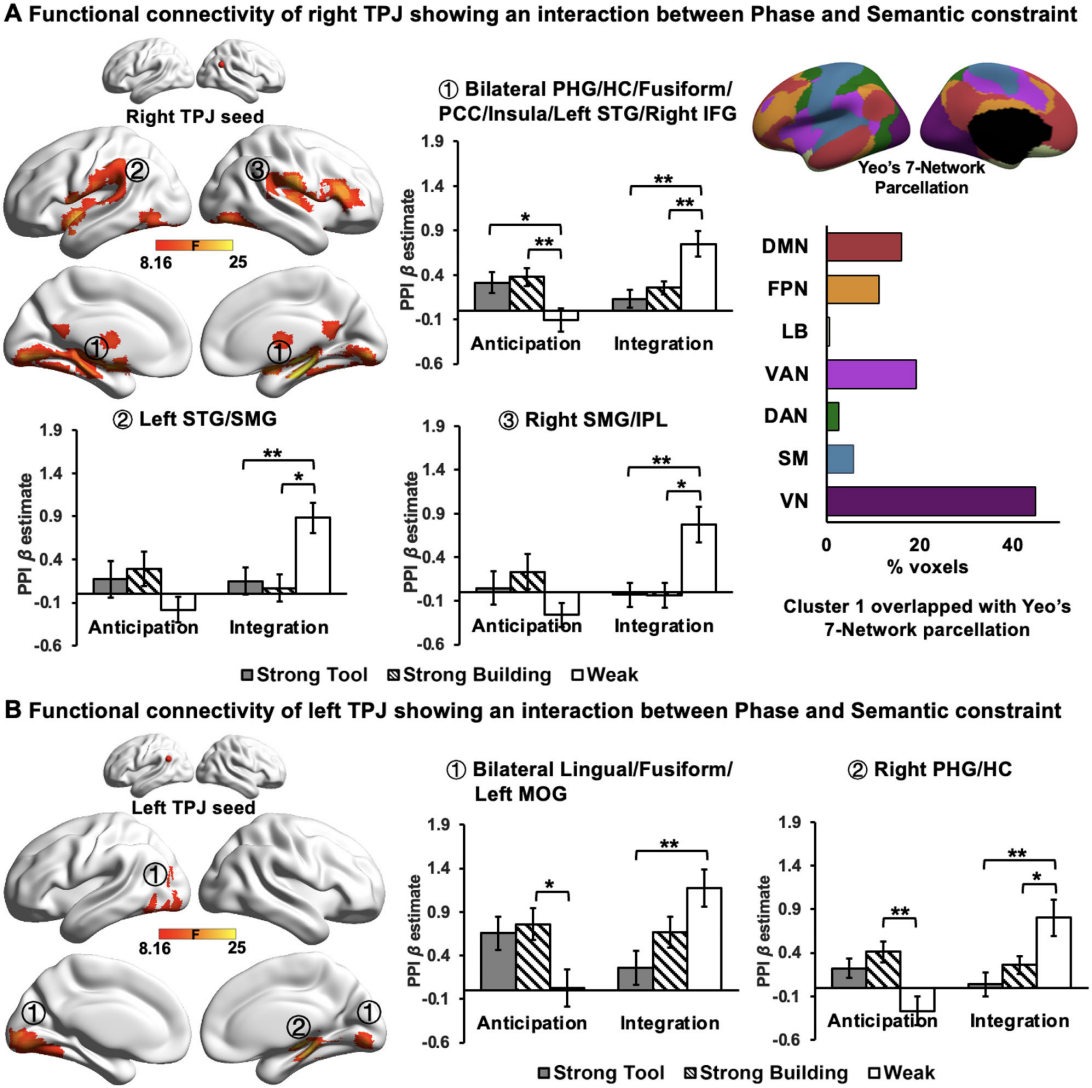
Functional connectivity seeding from the (***A***) right TPJ and (***B***) left TPJ showing an interaction between the processing phase (anticipation vs integration) and semantic constraint (Strong Building vs Strong Tool vs Weak). The bar charts plot the mean PPI *β* values (i.e., representing the strength of functional connectivity between the TPJ seeds and each identified cluster). Error bars depict the standard error of the mean. The detailed statistical values for post hoc *t* test can be found in tables in Extended Data Figure 3-1. The bar chart in the top right panel shows the percentage of overlap between Cluster 1 and Yeo's seven-network parcellation when seeding from the right TPJ. DMN, default mode network; FPN, frontoparietal control network; LB, limbic network; VAN, ventral attention network; DAN, dorsal attention network; SM, somatomotor network; VN, visual network. All statistics were corrected for multiple comparisons. * indicates Bonferroni-corrected *p* < 0.05, ** indicates Bonferroni-corrected *p* < 0.01, and *** indicates Bonferroni-corrected *p* < 0.001. PHG, parahippocampal gyrus; HC, hippocampus; PCC, posterior cingulated cortex; STG, superior temporal gyrus; IFG, inferior frontal gyrus; SMG, supramarginal gyrus; IPL, inferior parietal lobule; MOG, middle occipital gyrus.

For the right TPJ seed, an interaction between processing phase (anticipation vs integration) and semantic constraint (Strong Building vs Strong Tool vs Weak) was observed in three clusters (Fig. 3*A*): Cluster 1 included the right IFG and bilateral parahippocampal gyrus (PHG), hippocampus (HC), fusiform gyrus, posterior cingulate cortex (PCC), and insula, extending to left superior temporal gyrus (STG), Cluster 2 included the left STG and left SMG, and Cluster 3 included the right SMG and inferior parietal lobule (IPL). To better understand the nature of this connectivity effect, we extracted PPI *β* estimates for each condition in each identified cluster (Fig. 3*A*, bar charts). The post hoc *t* tests revealed that the functional connectivity of the right TPJ with regions within Cluster 1 was significantly enhanced in the Strong versus Weak semantic constraint condition during the anticipatory phase, while the connectivity of the right TPJ with Cluster 1, as well as its functional coupling to Clusters 2 and 3, was significantly reduced in the Strong relative to Weak semantic constraint condition during the integration phase (for detailed statistical reports, see Extended Data Fig. 3-1). The voxels within these identified clusters, particularly the largest Cluster 1 (Fig. 3*A*, bar chart in the top right panel), overlapped with visual, control networks [i.e., ventral attention network (VAN) and FPN], as well as part of DMN in the medial parietal and temporal gyrus (Table 2).

10.1523/JNEUROSCI.1404-24.2024.f3-1 Figure 3-1 Post-hoc paired *t*-tests of right and left TPJ connectivity patterns. Brain regions refer to the areas where peak coordinates are located. These *p* values were Bonferroni corrected for multiple statistical tests*.* * indicates *p* < 0.05, ** indicates *p* < 0.01, *** indicates *p* < 0.001. StrongT = Strong Tool; StrongB = Strong Building; PHG = Parahippocampal gyrus; SMG = Supramarginal gyrus, MOG = Middle occipital gyrus. Download Figure 3-1, DOC file.

These results suggest that when semantic constraints were strong and participants were able to predict upcoming semantic content, the functional coupling of the right TPJ to regions within DMN, control, and visual networks was higher during the anticipatory phase and weaker during the integration phase, compared with when semantic constraints were weak.

### Results for the left TPJ

For the left TPJ seed, an interaction between processing phase and semantic constraint was also observed in the lingual gyrus, fusiform gyrus, PHG, right HC and middle occipital gyrus (MOG; Fig. 3*B*). We extracted PPI *β* estimates for each condition in each identified cluster, and post hoc *t* tests revealed that the connectivity of the left TPJ with these clusters was higher in the Strong Building compared with Weak semantic constraint condition during the anticipatory phase. In contrast, during the integration phase, the visual connectivity of the left TPJ was significantly reduced in the Strong Tool compared with the Weak constraint condition, and the connectivity strength of the left TPJ with PHG/HC exhibited a reduction in both Strong constraint conditions relative to Weak constraint condition (Fig. 3*B*; for detailed statistical reports, see Extended Data Fig. 3-1). The voxels within these identified clusters were largely overlapping with the visual system, as well as part of the control and DMN systems (Table 2).

These results suggest that when semantic constraints were strong, the functional coupling of the left TPJ to the visual cortex was higher during the anticipatory phase, while this connectivity pattern was weaker during the integration phase, compared with when the semantic constraints were weak.

### Results for the left IFG

**Figure 4.**
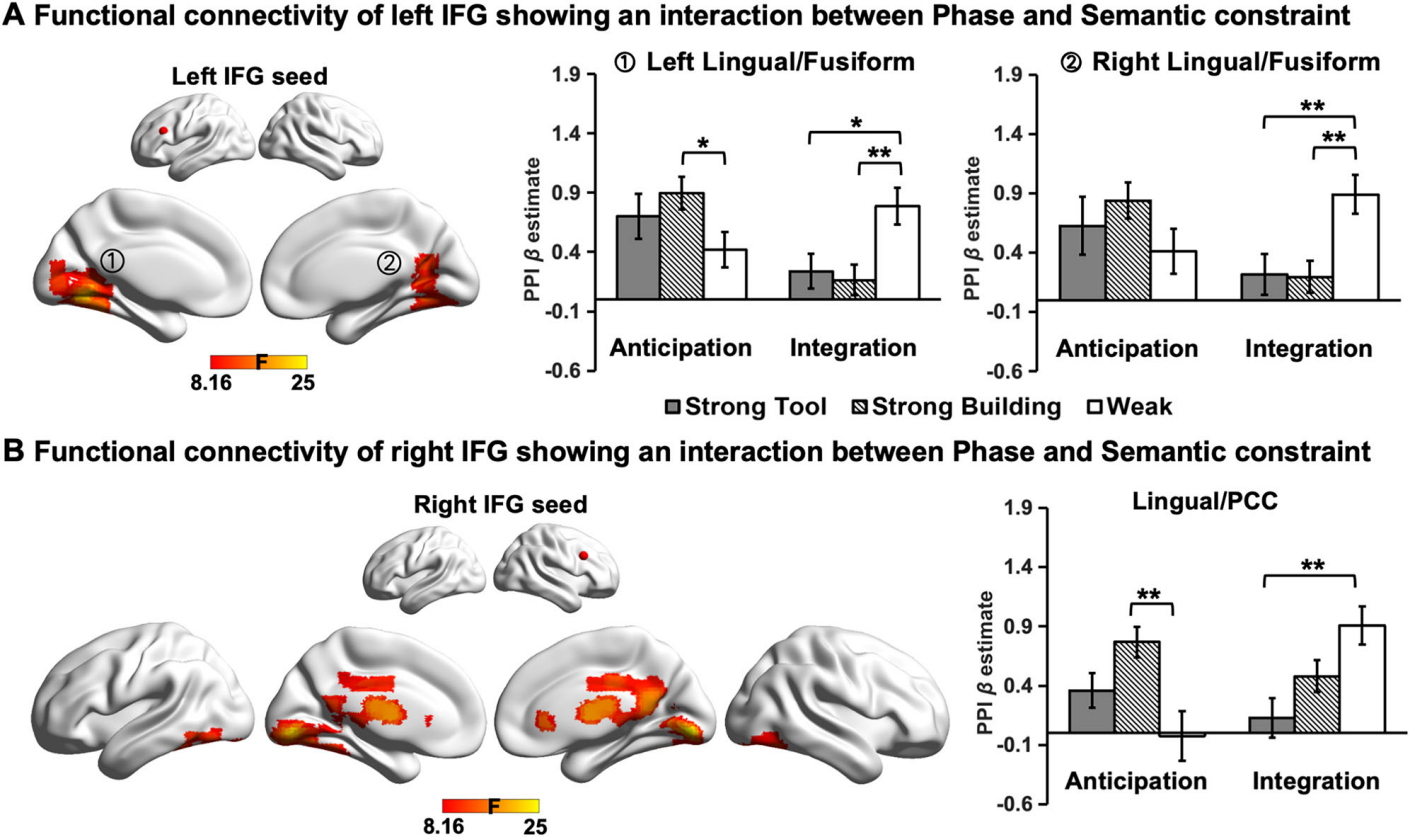
Functional connectivity seeding from the (***A***) left IFG and (***B***) right IFG seeds showing an interaction between the processing phase (anticipation vs integration) and semantic constraint (Strong Building vs Strong Tool vs Weak). The bar charts plot the mean PPI *β* values (i.e., representing the strength of functional connectivity between the IFG seeds and each identified cluster). Error bars depict the standard error of the mean. The detailed statistical values for post hoc *t* test can be found in the table in Extended Data Figure 4-1. All statistics were corrected for multiple comparisons. * indicates Bonferroni-corrected *p* < 0.05, and ** indicates Bonferroni-corrected *p* < 0.01. PCC, posterior cingulated cortex.

For the left IFG seed, we found an interaction between processing phase and semantic constraint in the bilateral lingual, fusiform gyrus, and PCC (Fig. 4*A*). Of the voxels within these identified clusters that fell within the seven large-scale networks defined by Yeo et al. (2011), 97.66% fell within the visual network (Table 2). To better understand the nature of this interaction, we extracted PPI *β* estimates for each condition in each cluster (Fig. 4*A*). Post hoc *t* tests revealed that during the integration phase, the functional connectivity of the left IFG with these two clusters was stronger in the Weak than that in the Strong semantic constraint conditions; in contrast, during the anticipatory phase, the functional connectivity of the left IFG with Cluster 1 was stronger in the Strong Building than that in the Weak semantic constraint condition (for detailed statistical reports, see Extended Data Fig. 4-1).

10.1523/JNEUROSCI.1404-24.2024.f4-1 Figure 4-1 Post-hoc paired *t*-tests of left and right IFG connectivity patterns. Brain regions refer to the areas where peak coordinates are located. These *p* values were Bonferroni corrected for multiple statistical tests. * indicates *p* < 0.05, ** indicates *p* < 0.01. StrongT = Strong Tool; StrongB = Strong Building. Download Figure 4-1, DOC file.

These results suggest that the functional coupling of the left IFG with the visual cortex was stronger in the Strong Building condition during the anticipatory phase and in the Weak semantic constraint condition during the integrative processing.

### Results for the right IFG

For the right IFG seed, an interaction between processing phase and semantic constraint was also observed in the bilateral lingual gyrus, PCC, and inferior temporal gyrus (Fig. 4*B*). The voxels within this identified cluster mainly overlapped with the visual network and DMN (Table 2). To better understand the nature of this interaction, we extracted PPI *β* estimates for each condition in the identified cluster. Post hoc *t* tests revealed that the connectivity of the right IFG with this cluster was stronger in the Strong Building compared with Weak semantic constraint condition during the anticipatory phase; in contrast, during the integration phase, this connectivity was weaker in the Strong Tool compared with Weak semantic constraint condition (Fig. 4*B*; for detailed statistical reports, see Extended Data Fig. 4-1).

These results suggest that when semantic constraints were strong, the functional coupling of the right IFG to the visual cortex and DMN in the medial parietal area was higher during the anticipatory phase, while this connectivity pattern was weaker during the integration phase, compared with when the semantic constraint was weak.

### Common interaction effects for TPJ and IFG

**Figure 5.**
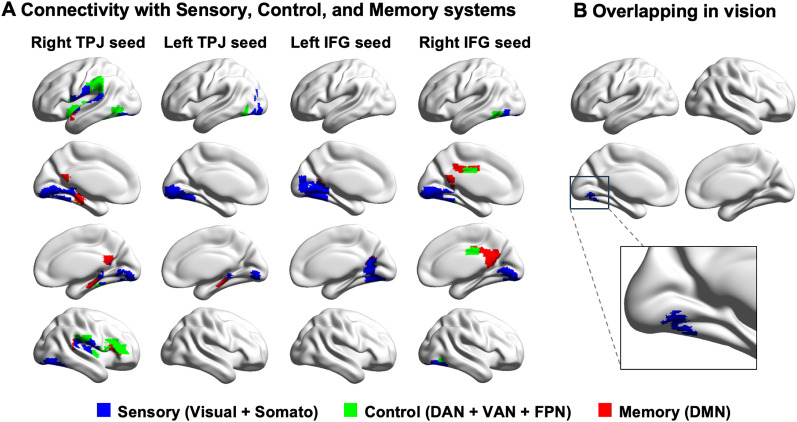
Overlapping connectivity of IFG and TPJ seeds*.* ***A***, The regions that showed an interaction effect (between the processing phase and semantic constraint) when seeding from IFG and TPJ seeds overlapped with sensory, control, and memory networks. ***B***, These interaction effects overlapped in the visual cortex, which was obtained by identifying the voxels in the four cluster-corrected connectivity maps showing an interaction effect with *F*-values > 8.17. Somato, somatomotor network; VAN, ventral attention network; FPN, frontoparietal control network; DMN, default mode network.

Our study aims to examine the connectivity patterns of frontoparietal sites that support semantic prediction and integration during predictive language comprehension. We found an interaction effect between processing phase (anticipation vs integration) and semantic constraint (Strong Building vs Strong Tool vs Weak) in visual, control, and memory systems when seeding from the right TPJ and mainly in the visual system when seeding from the left TPJ and IFG (for a summarization of these effects, see Fig. 5*A*).

To establish whether these effects draw on the same region of the visual cortex, we compared these four connectivity maps. We found that they overlapped in the left lingual gyrus and fusiform gyrus (Fig. 5*B*). Moreover, 100% of the voxels in this overlapping cluster fell within the visual network. This indicates that both IFG and TPJ seeds exhibited stronger functional coupling with the visual cortex when anticipating semantic information in highly predictable contexts and when the semantic constraint was weak during the integration phase. This suggests that stronger visual functional coupling of TPJ and IFG is important for supporting both top-down semantic prediction in highly predictive contexts and bottom-up semantic integration when the upcoming semantic information cannot be predicted.

## Discussion

Contemporary cognitive neuroscience has shown that predictive language comprehension is subserved by widely distributed, yet hierarchical, brain regions, with the frontoparietal cortex including TPJ and IFG situated at the top of this hierarchical neural network (Schmitt et al., 2019; Caucheteux et al., 2023). However, it remains unclear whether and how these higher-order predictive regions interact with other brain systems to support this semantic predictive processing during language comprehension. To this end, we manipulated the strength of semantic constraint provided by the sentential context on upcoming semantic content (Strong vs Weak), with a focus on both prediction generation (i.e., before the onset of targets) and integration processes (i.e., after the onset of the targets). We found that, in strong semantic constraint contexts, the connectivity of the right TPJ with PHG/PCC in DMN, IFG within the control network, and visual cortex was stronger during anticipation. This connectivity pattern, along with the right TPJ-to-SMG (in control network) connectivity, was weaker during the integration phase, i.e., when the predicted semantic contents appeared. A similar pattern was also observed in the visual coupling of the left TPJ and bilateral IFG, and in the right IFG-to-PCC connectivity, with stronger connectivity during anticipation and weaker connectivity during integration in a highly predictive context. These effects overlapped in the visual cortex, indicating the importance of visual coupling of both TPJ and IFG in supporting semantic prediction. These findings suggest that semantic predictive processing relies upon the diverse functional connectivity of the frontoparietal cortex with memory, control, and visual systems.

Our study builds on existing evidence that both IFG and TPJ within the frontoparietal cortex play a crucial role in active anticipation of upcoming semantic representations (Shain et al., 2020; Masina et al., 2022; Shao et al., 2022; Caucheteux et al., 2023). IFG has been considered a core higher-order cortical region implicated in predictive processing (Caucheteux et al., 2023), and it has been demonstrated to be involved in sentence comprehension and controlled semantic processing, including semantic retrieval/selection and semantic binding (Badre and Wagner, 2002; Davey et al., 2016; Chiou et al., 2018; Zhang et al., 2021; Zhang et al., 2022). TPJ is also thought to play a key role in predictive processing, which might relate to its contribution to the integration of diverse information and updating of internal contextual representations (Price et al., 2015; Margulies et al., 2016; Lanzoni et al., 2020; Smallwood et al., 2021; Masina et al., 2022). Our findings complement these studies by highlighting the important roles that IFG and TPJ play in semantic predictive processing through the lens of their functional connectivity with a set of brain areas within hierarchical predictive neural networks (as shown in Fig. 1*A*).

Our findings suggest that diverse patterns of frontoparietal connectivity support top-down semantic prediction based on available contextual information. We established that, in highly constraining semantic contexts, IFG and TPJ exhibited stronger connectivity with widely distributed brain areas in the IFG, visual cortex, PCC, and PHG during prediction generation. The brain regions within DMN and control networks are essential components supporting semantic prediction (Bonhage et al., 2015; Willems et al., 2016; Carter et al., 2019; Siman-Tov et al., 2019; Shao et al., 2022), and they have been repeatedly linked to high-level memory representation and information integration, attentional control, and other high-level executive functions (Gilbert and Burgess, 2008; Ahrens et al., 2019; Allan et al., 2020; Lanzoni et al., 2020; Nee, 2021; Smallwood et al., 2021). To support predictive processing, the brain must constantly integrate and update internal representations of sentential context as sensory inputs unfold over time, while simultaneously employing control processes to allocate attention and constrain semantic retrieval to the pertinent context. Our results resonate with these previous studies, highlighting the importance of brain regions within DMN and control networks in semantic predictive processing. Importantly, our results also extend these insights by demonstrating how the higher-order frontoparietal cortex, via its wide functional connections with these brain areas, supports semantic prediction. That is, the diverse connectivity patterns of the frontoparietal cortex might support the integration of sensory inputs to build up a supportive context, upon which upcoming semantic content can be predicted and preactivated to facilitate comprehension.

Intriguingly, our findings revealed the importance of visual coupling of frontoparietal cortices in semantic prediction, as evidenced by a common pattern of stronger visual connectivity of TPJ and IFG during prediction generation. The visual cortex feeds sensory inputs forward to higher-level predictive cortices, such as IFG, for higher-order computation and unification (Hagoort, 2005, 2013). It also plays a key role in representing the visual features of semantic knowledge (Lambon Ralph et al., 2017), which consequently supports the retrieval of semantic knowledge through visual imagery (Peelen and Caramazza, 2012; Bergmann et al., 2016; Borghesani et al., 2016; Dijkstra et al., 2017). The visual cortex is also involved in visual imagery during language comprehension (Just et al., 2004). The activation of the visual cortex can be modulated by top-down control processes within left IFG to facilitate efficient semantic retrieval (Bar, 2007; Zhang et al., 2021). Similarly, the stronger visual coupling of the frontoparietal cortex during semantic prediction might reflect the modulation of the extent to which sensory input can be passed up to higher-level cortices in the predictive processing hierarchy or preactivated semantic imagery from contextual information. While these potential explanations cannot be separated in the current study, our findings highlight the importance of the communication of the higher-order frontoparietal cortex with the sensory system in supporting semantic prediction.

Our study shows how predictive coding theory may operate in a visual semantic context to efficiently understand the meaning of written words, and our data support the suggestion that prediction generation and updating of an internal model are performed by the same hierarchical cortical network (K. Friston, 2005, 2010; K. J. Friston et al., 2017). These two phases of predictive language comprehension were found to rely on similar frontoparietal connectivity, with the strength of this pattern changing across phases, reflecting a reduction in connectivity when predicted semantic contents appeared, and an increase when current sensory input cannot be predicted from previous contexts. In situations in which upcoming information can be predicted based on available contextual information, diverse frontoparietal connectivity patterns support prediction generation; when these predictions are confirmed by subsequent sensory inputs, the demand on the communication between higher-order frontoparietal cortex and bottom-up regions is reduced as feed-forward propagation is suppressed (K. Friston, 2010). However, in an unpredictable situation, before the appearance of critical targets, weaker connectivity might suppress feed-forward processing, preventing the generation of semantic predictions that might be irrelevant, or even distracting; instead, after the appearance of these targets, stronger connectivity might support the integration of these inputs with the previous contextual information to achieve coherent comprehension. This flexible connectivity based on the availability of contextual information might be key to the efficiency of language comprehension.

Although our study provides important insights into how the higher-order frontoparietal cortex interacts with other brain systems to support semantic prediction, it also leaves open some important questions. First, we found these connectivity patterns can support both top-down prediction generation and bottom-up integration phases. The key differences between these two processing phases concern the timing and direction of connectivity between the frontoparietal cortex and other brain systems, which cannot be readily separated using fMRI data. To address this issue, future research employing high temporal resolution magnetoencephalography could potentially establish how information is transferred across these systems. Second, it is important to acknowledge the inherent differences in category variability between the Strong and Weak constraint conditions, as the Strong constraint conditions consistently limited target nouns to tool or building-related categories, while the Weak constraint conditions encompassed a wider range of categories, including sports, food, and daily life objects. Although this category variability might influence the observed effects, we ensured that the target nouns were always the best completion of the preceding context in all conditions to prevent semantic violations. Future research can eliminate the potential impact of this variability by incorporating a broader range of semantic categories of strong constraint conditions. Finally, it would also be useful to establish whether the identified connectivity patterns within the hierarchical predictive neural networks can predict individual differences in semantic prediction.

In conclusion, the diverse connectivity patterns of the right TPJ and right IFG with cognitive control, memory, and sensory systems, as well as visual connectivity of the left TPJ and bilateral IFG, support both top-down semantic prediction and bottom-up integration during semantic predictive processing. This flexible connectivity of the higher-order frontoparietal cortex with other brain systems contributes to our efficient language comprehension.
