# Supplementary material for: Diverse Frontoparietal Connectivity Supports Semantic Prediction and Integration in Sentence Comprehension
Source: J Neurosci. 2024 Nov 12;45(5):e1404242024. doi: 10.1523/JNEUROSCI.1404-24.2024 (PMC11780348; doi:10.1523/JNEUROSCI.1404-24.2024)
Supplement: Figure 2-1 — Cloze probability (CP) of the Critical Nouns (and Other Completed Words) in the three Experimental Conditions. “Critical nouns” indicate the critical nouns used in the experimental sentences of corresponding experimental condition, “tool-nouns” indicate all of the completed nouns that belong to the tool category, and “building-nouns” indicate all of the completed nouns that belong to the building category. “CP of pronoun + verb” indicates the averaged CP of the critical verbs and the pronouns immediately preceding these verbs. “N.A.” indicates that the corresponding CP value is not applicable, as the pronoun and verb have already been presented at the “Preceding Critical Noun” position. For the Weak-constraint condition, the critical nouns were the best completion in Test 2. Download Figure 2-1, DOC file. [file jneuro-45-e1404242024-s001.doc]

|  |  | Preceding Critical Verb (Test 1) | |  | Preceding Critical Noun (Test 2) | |
| --- | --- | --- | --- | --- | --- | --- |
| Conditions | | M | SD |  | M | SD |
| Tool | CP of critical nouns | 42.46% | 18.63% |  | 85.13% | 11.13% |
|  | CP of all tool-nouns | 49.35% | 18.48% |  | 90.95% | 8.93% |
|  | CP of “pronoun+verb” | 15.19% | 13.17% |  | N.A. | N.A. |
|  |  |  |  |  |  |  |
| Building | CP of critical nouns | 34.91% | 20.49% |  | 80.82% | 8.67% |
|  | CP of all building-nouns | 42.03% | 22.22% |  | 85.99% | 8.78% |
|  | CP of “pronoun+verb” | 16.38% | 10.75% |  | N.A. | N.A. |
|  |  |  |  |  |  |  |
| Weak | CP of best completion | 20.04% | 11.5% |  | 22.63% | 6.97% |
|  | CP of all tool-nouns | 2.16% | 5.35% |  | 5.17% | 9.01% |
|  | CP of all building-nouns | 1.72% | 4.39% |  | 7.97% | 10.55% |
|  | CP of “pronoun+verb” | 7.54% | 8.94% |  | N.A. | N.A. |
