# Supplementary material for: Diverse Frontoparietal Connectivity Supports Semantic Prediction and Integration in Sentence Comprehension
Source: J Neurosci. 2024 Nov 12;45(5):e1404242024. doi: 10.1523/JNEUROSCI.1404-24.2024 (PMC11780348; doi:10.1523/JNEUROSCI.1404-24.2024)
Supplement: Figure 2-2 — The linguistic properties of the critical nouns in the end of sentences. Download Figure 2-2, DOC file. [file jneuro-45-e1404242024-s002.doc]

| Linguistic Properties | Strong  Tool | | Strong Building | | Weak | | One-way ANOVA | |
| --- | --- | --- | --- | --- | --- | --- | --- | --- |
|  | M | SD | M | SD | M | SD | *F* | *p* |
| Imageability | 6.63 | 0.17 | 5.59 | 0.46 | 5.88 | 0.85 | 22.83 | <0.001 |
| Operability | 6.47 | 0.31 | 2.25 | 0.37 | 4.87 | 1.15 | 284.70 | <0.001 |
| Word Frequency | 1.70 | 0.59 | 2.00 | 0.70 | 2.01 | 0.79 | 1.68 | 0.19 |
| Stroke Number | 19.62 | 8.42 | 18.79 | 6.64 | 20.52 | 5.23 | 0.46 | 0.64 |
