# Supplementary material for: Diverse Frontoparietal Connectivity Supports Semantic Prediction and Integration in Sentence Comprehension
Source: J Neurosci. 2024 Nov 12;45(5):e1404242024. doi: 10.1523/JNEUROSCI.1404-24.2024 (PMC11780348; doi:10.1523/JNEUROSCI.1404-24.2024)
Supplement: Figure 3-1 — Post-hoc paired t-tests of right and left TPJ connectivity patterns. Brain regions refer to the areas where peak coordinates are located. These p values were Bonferroni corrected for multiple statistical tests. * indicates p < 0.05, ** indicates p < 0.01, *** indicates p < 0.001. StrongT = Strong Tool; StrongB = Strong Building; PHG = Parahippocampal gyrus; SMG = Supramarginal gyrus, MOG = Middle occipital gyrus. Download Figure 3-1, DOC file. [file jneuro-45-e1404242024-s003.doc]

| Right TPJ seed  /Brain Region | Contrast | Anticipatory Phase | |  | Integration Phase | |
| --- | --- | --- | --- | --- | --- | --- |
|  |  | *t* | *p* |  | *t* | *p* |
| Right PHG | StrongT *vs.* Weak | 2.68 | 0.042* |  | -4.45 | 0.001** |
| /Fusiform | StrongB *vs.* Weak | 3.67 | 0.004** |  | -3.3 | 0.01** |
|  | StrongT *vs.* StrongB | -0.52 | 1 |  | -1.06 | 0.899 |
| Left SMG | StrongT *vs.* Weak | 1.43 | 0.503 |  | -3.88 | 0.003** |
|  | StrongB *vs.* Weak | 2.17 | 0.125 |  | -3.19 | 0.013* |
|  | StrongT *vs.* StrongB | -0.47 | 1 |  | 0.34 | 1 |
| Right SMG | StrongT *vs.* Weak | 1.45 | 0.486 |  | -4.33 | 0.001** |
|  | StrongB *vs.* Weak | 2.26 | 0.103 |  | -2.85 | 0.029* |
|  | StrongT *vs.* StrongB | -0.71 | 1 |  | 0.02 | 1 |

| Left TPJ seed  /Brain Region | Contrast | Anticipatory Phase | |  | Integration Phase | |
| --- | --- | --- | --- | --- | --- | --- |
|  |  | *t* | *p* |  | *t* | *p* |
| Left MOG | StrongT *vs.* Weak | 2.42 | 0.075 |  | -3.89 | 0.003** |
| /Lingual | StrongB *vs.* Weak | 2.98 | 0.022* |  | -1.73 | 0.298 |
|  | StrongT *vs.* StrongB | -0.44 | 1 |  | -1.77 | 0.272 |
| Right | StrongT *vs.* Weak | 2.24 | 0.108 |  | -3.39 | 0.009** |
| Hippocampus | StrongB *vs.* Weak | 4.15 | 0.002** |  | -2.82 | 0.031* |
|  | StrongT *vs.* StrongB | -1.21 | 0.716 |  | -1.28 | 0.645 |
