# Supplementary material for: Diverse Frontoparietal Connectivity Supports Semantic Prediction and Integration in Sentence Comprehension
Source: J Neurosci. 2024 Nov 12;45(5):e1404242024. doi: 10.1523/JNEUROSCI.1404-24.2024 (PMC11780348; doi:10.1523/JNEUROSCI.1404-24.2024)
Supplement: Figure 4-1 — Post-hoc paired t-tests of left and right IFG connectivity patterns. Brain regions refer to the areas where peak coordinates are located. These p values were Bonferroni corrected for multiple statistical tests. * indicates p < 0.05, ** indicates p < 0.01. StrongT = Strong Tool; StrongB = Strong Building. Download Figure 4-1, DOC file. [file jneuro-45-e1404242024-s004.doc]

| Seed/  Brain Region | Contrast | Anticipatory Phase | |  | Integration Phase | |
| --- | --- | --- | --- | --- | --- | --- |
|  |  | *t* | *p* |  | *t* | *p* |
| Left IFG seed |  |  |  |  |  |  |
| Left Lingual | StrongT vs. Weak | 1.19 | 0.74 |  | -3.27 | 0.011* |
|  | StrongB vs. Weak | 2.66 | 0.044* |  | -3.45 | 0.007** |
|  | StrongT vs. StrongB | -0.74 | 1 |  | 0.39 | 1 |
| Right Fusiform | StrongT vs. Weak | 0.74 | 1 |  | -3.72 | 0.004** |
|  | StrongB vs. Weak | 2.22 | 0.112 |  | -3.58 | 0.005** |
|  | StrongT vs. StrongB | -0.66 | 1 |  | 0.11 | 1 |
| Right IFG seed |  |  |  |  |  |  |
| Right Lingual | StrongT vs. Weak | 2.11 | 0.14 |  | -3.84 | 0.003** |
|  | StrongB vs. Weak | 3.79 | 0.003** |  | -2.18 | 0.12 |
|  | StrongT vs. StrongB | -2.54 | 0.056 |  | -1.8 | 0.257 |
